# Dexamethasone mimics aspects of physiological acclimatization to 8 hours of hypoxia but suppresses plasma erythropoietin

**DOI:** 10.1152/japplphysiol.01414.2012

**Published:** 2013-02-07

**Authors:** Chun Liu, Quentin P. P. Croft, Swati Kalidhar, Jerome T. Brooks, Mari Herigstad, Thomas G. Smith, Keith L. Dorrington, Peter A. Robbins

**Affiliations:** Department of Physiology, Anatomy & Genetics, University of Oxford, Oxford, United Kingdom

**Keywords:** dexamethasone, oxygen, erythropoietin, pulmonary circulation, ventilation, cardiac output

## Abstract

Dexamethasone ameliorates the severity of acute mountain sickness (AMS) but it is unknown whether it obtunds normal physiological responses to hypoxia. We studied whether dexamethasone enhanced or inhibited the ventilatory, cardiovascular, and pulmonary vascular responses to sustained (8 h) hypoxia. Eight healthy volunteers were studied, each on four separate occasions, permitting four different protocols. These were: dexamethasone (20 mg orally) beginning 2 h before a control period of 8 h of air breathing; dexamethasone with 8 h of isocapnic hypoxia (end-tidal Po_2_ = 50 Torr); placebo with 8 h of air breathing; and placebo with 8 h of isocapnic hypoxia. Before and after each protocol, the following were determined under both euoxic and hypoxic conditions: ventilation; pulmonary artery pressure (estimated using echocardiography to assess maximum tricuspid pressure difference); heart rate; and cardiac output. Plasma concentrations of erythropoietin (EPO) were also determined. Dexamethasone had no early (2-h) effect on any variable. Both dexamethasone and 8 h of hypoxia increased euoxic values of ventilation, pulmonary artery pressure, and heart rate, together with the ventilatory sensitivity to acute hypoxia. These effects were independent and additive. Eight hours of hypoxia, but not dexamethasone, increased the sensitivity of pulmonary artery pressure to acute hypoxia. Dexamethasone, but not 8 h of hypoxia, increased both cardiac output and systemic arterial pressure. Dexamethasone abolished the rise in EPO induced by 8 h of hypoxia. In summary, dexamethasone enhances ventilatory acclimatization to hypoxia. Thus, dexamethasone in AMS may improve oxygenation and thereby indirectly lower pulmonary artery pressure.

normal physiological responses to sustained hypoxia involve both an increase in pulmonary ventilation and an increase in pulmonary artery pressure, the latter arising from hypoxic pulmonary vasoconstriction. The first of these responses may be observed as an advantageous acclimatization that limits the degree of oxyhemoglobin desaturation of systemic arterial blood; the second of these responses appears to have no benefit, while predisposing to pulmonary edema and right-sided heart failure. Abnormal responses to sustained hypoxia include both the relatively common acute mountain sickness (AMS) together with the rarer diseases of high-altitude pulmonary edema and high-altitude cerebral edema.

Dexamethasone, a potent synthetic glucocorticoid, has been used extensively to treat high-altitude illness. In AMS, dexamethasone noticeably reduces the symptoms and signs of the illness, and it has been used both prophylactically and therapeutically in this condition (11, 12, 18, 38). In treating AMS, dexamethasone has been noted to increase oxyhemoglobin saturation when compared with control (12). In patients who have previously been shown to be susceptible to high-altitude pulmonary edema and who develop very high pulmonary arterial pressures on exposure to hypoxia, dexamethasone has been shown to reduce the rise in pulmonary arterial pressure when such patients are reexposed to the hypoxia of high altitude (13, 30).

Given the use of dexamethasone to prevent or treat high-altitude disease, the purpose of this study was to define the actions of dexamethasone on the early stages of ventilatory acclimatization to hypoxia in normal individuals. We hypothesized that dexamethasone might enhance the beneficial ventilatory acclimatization to hypoxia while obtunding the disadvantageous pulmonary vascular response to hypoxia. We examined this hypothesis by measuring the effects of dexamethasone on respiratory and cardiovascular responses to brief episodes (20 min) of hypoxia with and without an immediately preceding period of 8 h of hypoxia that previously has been shown to generate a marked degree of acclimatization to hypoxia; we designed the study to avoid any confounding effects of hypocapnia. We further examined the effect of dexamethasone on the response to 8 h of hypoxia of the hypoxia-inducible factor (HIF)-regulated product erythropoietin (EPO).

## METHODS

### Participants

Eight healthy volunteers (5 men, 3 women) participated in the study with an average age of 24 ± 3 yr, height 172 ± 8 cm, and weight 63 ± 14 kg (mean ± SD). Each volunteer gave informed consent before participating. The study was approved by the Central Oxford Research Ethics Committee and performed in accordance with the Declaration of Helsinki. Each volunteer visited the laboratory twice before undertaking any of the main experimental protocols to become familiar with the laboratory and its procedures, and for confirmation of suitability for echocardiographic measurement of tricuspid regurgitation.

### Protocols

All participants undertook four different protocols on four different days. The four protocols used were as follows: *1)* in *Protocol C-A* (control drug-air), placebo tablets were given and volunteers were exposed to an 8-h period of air breathing; *2)* in *Protocol C-H* (control drug-hypoxia), placebo tablets were given and exposure was to 8 h of isocapnic hypoxia; *3)* in *Protocol D-A* (dexamethasone-air), dexamethasone tablets were given and exposure was to 8 h of air breathing; and *4)* in *Protocol D-H* (dexamethasone-hypoxia), dexamethasone tablets were given and exposure was to 8 h of isocapnic hypoxia. The protocols were carried out according to a block-randomized order determined before recruitment. There was at least a 1-wk interval between two successive protocols. Female subjects were studied only during the first 2 wk of their menstrual cycle unless they were taking a contraceptive pill, because levels of circulating progesterone are known to affect aspects of respiratory control such as CO_2_ sensitivity (9).

In all protocols, participants reported to the laboratory and took the first dose (10 mg) of dexamethasone/placebo tablets at 7:00 am and then rested for 2 h. We chose a slightly higher dose than the 8 mg, 12-hourly dose used by others over longer periods, because no time was available for loading (13, 30). A blood sample was then collected and end-tidal Pco_2_ (Pet_CO_2__) was measured. We term this measurement the normal Pet_CO_2__ for the protocol. Participants were then exposed to acute isocapnic hypoxia (see below). Ventilation (V̇e), heart rate (HR), cardiac output (CO), systemic arterial pressure (SAP), and the maximum systolic pressure difference across the tricuspid valve (ΔPmax) were determined during these acute hypoxic exposures. We term all of these AM measurements. After the AM measurements, participants took a 30-min break and then entered a purpose-built chamber to be exposed to either 8 h of isocapnic hypoxia or 8 h of air breathing. The experimental procedure following these 8-h exposures was identical to that constituting the AM measurements. We term all of the measurements made at this stage PM measurements.

#### Control of End-Tidal Gases and Respiratory Measurement during Acute Hypoxic Exposures

In the acute hypoxic exposures, participants' end-tidal Po_2_ (Pet_O_2__) was held at 100 Torr for the first 10 min, then at 50 Torr for 20 min, and finally at 100 Torr again for 10 min. Pet_CO_2__ was held constant at 1.5 Torr above each participant's normal value throughout. During exposures to acute hypoxia, volunteers lay in a left-lateral position on a couch and breathed through a mouthpiece with the nose occluded. Ventilatory volumes were measured by a turbine volume-measuring device, and flows by a pneumotachograph, both in series with the mouthpiece. Respired gases were sampled via a fine catheter close to the mouth and analyzed continuously for Pco_2_ and Po_2_ by mass spectrometry. A pulse oximeter was used to monitor arterial oxyhemoglobin saturation. Inspired and expired volumes, end-inspiratory and end-expiratory Pco_2_ and Po_2_, and saturation were detected in real time by a computer and logged breath by breath. A dynamic end-tidal forcing system (36) was used to control the end-tidal gases in the manner required for the determination of the acute hypoxic ventilatory response. Before the start of each experiment, a cardiorespiratory model was used to construct a forcing function that contained the breath-by-breath values for inspiratory Pco_2_ and Po_2_ predicted to produce the desired end-tidal sequences. During the experiment, a computer-controlled gas-mixing system was used to generate this sequence in a modified manner. The modifications resulted from feedback control on the basis of deviations of the measured values for Pet_CO_2__ and Pet_O_2__ from their desired values.

#### Measurements of pulmonary blood pressure, CO, and SAP.

A standard technique was used for this measurement, as described in detail in a previous study from our laboratory (1). The majority of people have detectable tricuspid valve regurgitation during systole. Doppler echocardiography is able to detect the presence of this jet of blood and measure the velocity with which it travels back into the right atrium. On the assumption that the flow within the jet may be regarded as steady, the Bernoulli equation may be used to calculate the maximum pressure difference between the right ventricle and right atrium (ΔPmax) from the density of blood (ρ) and the peak velocity of the jet (v). This gives the relationship ΔPmax = ρv^2^/2. Assuming right atrial pressure remains constant, changes in ΔPmax will be equal to changes in the peak systolic pulmonary arterial pressure (33).

Echocardiographic measurements were performed by using a Hewlett-Packard Sonos 5500 ultrasound machine with an S4 two-dimensional transducer (2–4 MHz). Heart rate and respiratory waveform were both recorded simultaneously. Cardiac output was obtained from flow through the aortic valve obtained from an apical five-chamber view as previously described (1). Systemic arterial blood pressure was measured using an automatic inflatable arm cuff manometer (Omron M5-1; Omron Healthcare UK, West Sussex, UK).

#### Eight-hour exposure to hypoxia or air.

The 8-h period of isocapnic hypoxic exposure was conducted by using a purpose-built chamber, which was large enough to allow subjects to lie, sit, or stand comfortably. The chamber was normobaric, and isocapnic hypoxia was achieved by enriching the chamber air with N_2_ and CO_2_. Respired gas was sampled via a fine nasal catheter at the opening of the participant's nostril. The samples were continually analyzed for Pet_CO_2__ and Pet_O_2__. Arterial oxyhemoglobin saturation was monitored by using a pulse oximeter. A computer sampled the Pet_CO_2__, Pet_O_2__, and saturation signals at a frequency of 50 Hz. It detected end-inspiratory and end-expiratory values for Pco_2_ and Po_2_ and recorded these breath by breath along with values for saturation. The desired values for Pet_CO_2__ and Pet_O_2__ were entered into the computer. The computer then automatically adjusted the composition of the gas in the chamber every 5 min, or at manually overridden intervals, to minimize the error between desired and actual values for the end-tidal gases. This system has been described in detail elsewhere (17). Participants' Pet_O_2__ was held at 50 Torr and Pet_CO_2__ was held constant at each participant's normal value throughout the 8-h isocapnic hypoxia exposure. In the protocols involving an 8-h period of air breathing, participants occupied the same chamber and experienced the same monitoring while the chamber was supplied with air. A second dose of control drug or dexamethasone was administrated 3 h after participants entered the chamber.

### Measurement of Plasma EPO Concentration

For each of the four protocols, venous blood was collected in tubes coated with ethylenediamine tetraacetic acid. AM and PM samples were taken, respectively, before and after the 8-h chamber exposures. After venepuncture, each sample was immediately centrifuged at 1,000 g for 15 min. Plasma was removed and stored at −80°C until analysis. The concentration of EPO was measured using an ELISA for human specimens (Quantikine IVD DEP00; R&D System, Abingdon, UK). The coefficient of variation is stated by the manufacturer to be ∼5% for measurements made on human plasma.

### Modeling of Hypoxic Ventilatory Responses

To obtain numerical values for the sensitivity of V̇e to the acute hypoxic exposure, a respiratory model was fitted to the data. The particular respiratory model employed was Model I of those described by Liang et al. (26). This model separates the total ventilation V̇e into a central, hypoxia-independent component, V̇c, and a peripheral, hypoxia-dependent component, V̇p, and may be written:

$$\begin{array}{c} \dot{V}\text{E}=\dot{V}\text{p}+\dot{V}\text{c}\\ \text{T}\text{p}\frac{\text{d}{\dot{V}}_{\text{E}}}{\text{d}t}+\dot{V}\text{p}=\text{G}\text{p}\cdot \left[100-S\left(t-\text{D}\text{p}\right)+\text{K}\text{p}\right]\\ \text{T}\text{h}\frac{\text{dG}\text{p}}{\text{d}t}+\text{G}\text{p}={\text{G}}_{100}-\text{Gh}\cdot \left[100-S\left(t-\text{D}\text{p}\right)\right] \end{array}$$

where Tp is the time constant for development of the peripheral response, Kp is the peripheral drive in the absence of hypoxia, Gp is the peripheral chemoreflex sensitivity, and *S* is the saturation function (39) calculated at time *t* delayed by the peripheral time delay, Dp. Th is the time constant for the development of hypoxic ventilatory decline (HVD), G_100_ is the steady-state chemoreflex sensitivity in the absence of HVD (i.e., following conditioning at 100% arterial oxyhemoglobin saturation), and Gh defines the magnitude of HVD as the ratio of the decrease in peripheral chemoreflex sensitivity to the decrease in *S*.

To allow for the autocorrelation that exists between measurements of ventilation on successive breaths, a model of the noise processes was fitted to the data in parallel with the fitting of the model of the ventilatory response to hypoxia (25). The model parameters were estimated by fitting the model to the data using a standard subroutine to minimize the sum of squares of the residuals (subroutine E04FDF; Numerical Algorithms Group, Oxford, UK).

### Modeling of Pulmonary Vascular, CO, and HR Responses to Acute Hypoxia

To quantify fully the pulmonary vascular and cardiovascular responses to acute hypoxia observed in this study, a mathematical model was fitted to the data. In this model, it was assumed that the dynamics of the response could be represented by a simple, linear, first-order process and that the changes in ΔPmax, CO, and HR could be related in a linear manner to changes in saturation, at least in the range of Po_2_ values studied. Given these assumptions, a simple difference equation may be written of the form:

$${\text{Y}}_{\left(t\right)}={\text{B}}_{100}+\text{G}\cdot \left(100-S_{\left(t\right)}\right)-\left[{\text{B}}_{100}+\text{G}\cdot \left(100-S_{\left(t\right)}\right)-{\text{Y}}_{\left(t-1\right)}\right]\cdot e^{\left(-\Delta \text{t}/\text{T}\right)}$$

where the difference between two successive time points (t) and (t-1) is Δt; *S* is the saturation (39); B_100_ is the steady-state value for ΔPmax, CO, and HR in the absence of hypoxia (i.e., when saturation is 100%); G is the sensitivity of ΔPmax, CO, and HR to a decrease in saturation; and T is the time constant for the response. The parameters of this model (B, G, T) were estimated using the function LSQNONLIN in Matlab (version 7.0; MathWorks, Natick, MA) to minimize the sum of squares of the residuals.

### Quantification of Systemic Cardiovascular Responses

The responses of the systemic circulation to acute hypoxia were not sufficiently well defined to quantify through a model-fitting process. Accordingly, simple averages of these responses were calculated under the conditions of euoxia and hypoxia.

### Data Analysis

To examine whether either 8 h of isocapnic hypoxia or administration of dexamethasone had any effect on the model parameters, the AM value for each subject was subtracted from the PM value and the values obtained were subjected to a one-sample *t*-test. Significance was assumed at *P* < 0.05.

To check whether any significant effects of either 8 h of isocapnic hypoxia or administration of dexamethasone were different from the control experiment (no hypoxia, no dexamethasone), ANOVA was used with a fixed factor of protocol and a random factor of volunteer. Significance was again assumed at *P* < 0.05.

## RESULTS

### Ventilatory Responses to Hypoxia

**Fig. 1.**
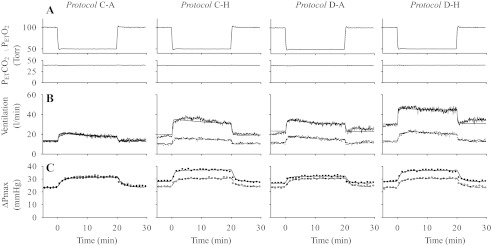
Mean values for *A*: end-tidal Po_2_ (Pet_O_2__) and end-tidal Pco_2_ (Pet_CO_2__) (15-s averages), *B*: ventilation (15-s averages), and *C*: maximum systolic pressure difference across the tricuspid valve during systole (ΔPmax) in the morning (AM measurements, open symbols or broken lines) and afternoon (PM measurements, closed symbols or continuous lines) in all four protocols. Lines through data for ventilation and ΔPmax indicate the fit of the relevant models to the data.

There were no significant differences between baseline values of Pet_CO_2__ in the four protocols. Figure 1*A* shows mean values of Pet_O_2__ and Pet_CO_2__ during the AM and PM acute hypoxic exposures for all four protocols. The acute hypoxic stimulus was generated accurately before and after the 8-h period of isocapnic hypoxia or the 8-h period of air breathing in each protocol. Pet_CO_2__ was held constant throughout. Overall, no discernible differences in gas control among the four protocols could be detected.

Figure 1*B* shows the mean responses for V̇e during the AM and PM acute hypoxic exposures for all four protocols. Table 1 lists the parameters obtained from the model fitting, together with results from the statistical comparisons. From these results, the V̇e in euoxia and acute hypoxia in the AM measurements did not differ significantly between protocols. Given that the first dose of dexamethasone or control drug had been administrated ∼2 h before this exposure to acute hypoxia, these results show that, acutely, dexamethasone had no effect on ventilation in either euoxia or acute hypoxia. Eight hours of isocapnic hypoxia and administration of dexamethasone, whether separately or in combination, both increased ventilation in both euoxia and acute hypoxia in the PM measurements relative to the AM measurements. For the parameters of the respiratory model, no significant differences were found between the PM and AM values for the control protocol (*Protocol C-A*). In contrast, there were significant increases in G_100_ and V̇c for *Protocol C-H*, *Protocol D-A*, and *Protocol D-H*. There were no significant differences among AM parameter values in the protocols. ANOVA confirmed that the increases in G_100_ and V̇c for *Protocol C-H*, *Protocol D-A*, and *Protocol D-H* were significantly different from the control protocol (*Protocol C-A*). For the baseline parameter V̇c, these effects appear to be approximately additive, but for the sensitivity G_100_, this does not appear to be the case. ANOVA did not detect any significant interaction between 8 h of isocapnic hypoxia and dexamethasone administration for any model parameter. Thus, isocapnic hypoxia and dexamethasone in combination induce no changes that significantly exceed the additive effects of the stimuli acting alone.

**Table 1.** Ventilatory responses to acute hypoxia: parameter values (mean ± SE) for respiratory model for the protocols with and without dexamethasone and with and without sustained hypoxia

	G_100_ l/min/%	V̇c liters/min	Tp seconds	Dp seconds	Gh liters/min/%^2^	Th seconds	Kp %
AM measurements							
*Protocol C-A*	0.71 ± 0.16	11.2 ± 1.1	17.2 ± 4.1	6.9 ± 2.4	0.025 ± 0.010	768 ± 112	1.7 ± 1.4
*Protocol C-H*	0.51 ± 0.07	8.1 ± 1.5	12.9 ± 4.8	6.2 ± 1.9	0.012 ± 0.005	511 ± 150	5.7 ± 4.2
*Protocol D-A*	0.58 ± 0.11	7.2 ± 1.3	11.4 ± 4.3	7.3 ± 1.7	0.020 ± 0.010	479 ± 112	8.6 ± 5.0
*Protocol D-H*	1.04 ± 0.24	8.9 ± 1.3	19.0 ± 4.0	7.0 ± 2.2	0.033 ± 0.012	552 ± 168	3.6 ± 2.4
PM measurements							
*Protocol C-A*	0.71 ± 0.2	11.6 ± 1.4	12.1 ± 3.8	5.9 ± 1.5	0.025 ± 0.011	471 ± 142	2.3 ± 1.3
*Protocol C-H*	1.31 ± 0.31	15.7 ± 1.5	17.5 ± 4.0	4.9 ± 2.2	0.030 ± 0.012	741 ± 97	1.6 ± 1.1
*Protocol D-A*	1.19 ± 0.25	19.7 ± 2.7	16.1 ± 4.2	9.5 ± 2.5	0.04 ± 0.012	471 ± 133	3.0 ± 2.5
*Protocol D-H*	1.59 ± 0.19	25.6 ± 3.5	19.3 ± 4.0	67 ± 2.5	0.036 ± 0.014	402 ± 144	2.0 ± 2.4
PM-AM measurements							
*Protocol C-A*	0.00 ± 0.11	0.3 ± 0.5	−5.1 ± 5.5	−1.0 ± 2.1	0.000 ± 0.010	−283 ± 90	0.6 ± 1.9
*Protocol C-H*	0.80 ± 0.32*	7.6 ± 1.4*	4.5 ± 5.9	−1.3 ± 1.4	0.018 ± 0.014	229 ± 163	−4.1 ± 4.7
*Protocol D-A*	0.61 ± 0.22*	12.5 ± 2.6*	4.6 ± 6.8	2.2 ± 3.3	0.019 ± 0.016	−8 ± 155	−5.6 ± 5.6
*Protocol D-H*	0.55 ± 0.16*	16.7 ± 3.4*	0.3 ± 6.3	−0.3 ± 2.5	0.001 ± 0.017	−150 ± 201	−1.6 ± 3.4

G_100_, steady-state chemoreflex sensitivity to hypoxia in the absence of any hypoxic ventilatory depression (arterial oxygen saturation is 100%); V̇c, hypoxia-independent (central chemoreflex) contribution to V̇e; Tp, time constant for the peripheral chemoreflex responses to hypoxia; Dp, time delay for the peripheral chemoreflex; Gh, sensitivity to hypoxic ventilatory decline, expressed as the ratio of the decrease in the sensitivity of the peripheral chemoreflex to the decrease in conditioning arterial oxygen saturation; Th, time constant associated with the development of hypoxic ventilatory decline; Kp, peripheral drive in the absence of hypoxia; *Protocol C-A*, control protocol without dexamethasone and with air breathing; *Protocol C-H*, protocol without dexamethasone and with sustained hypoxia; *Protocol D-A*, protocol with dexamethasone and with air breathing; *Protocol D-H*, protocol with dexamethasone and sustained hypoxia; AM, morning; PM, afternoon. One-sample *t*-test conducted on the differences between the morning and afternoon measurements.

* *P* < 0.05.

### Pulmonary Vascular Responses to Hypoxia

The responses of ΔPmax in each protocol are shown in Fig. 1*C*. The parameters obtained from fitting the cardiovascular model to ΔPmax together with results from the statistical comparisons are shown in Table 2. For the parameters from the model, no significant differences were found between the PM and AM values for *Protocol C-A*. In contrast, there were significant increases in baseline values of ΔPmax (B_ΔPmax_) for *Protocol C-H*, *Protocol D-A*, and *Protocol D-H*, while the sensitivity of ΔPmax to acute hypoxia (G_ΔPmax_) increased only in *Protocol C-H* and *Protocol D-H*. There was no interaction between 8 h of isocapnic hypoxia and the administration of dexamethasone on B_ΔPmax_ or G_ΔPmax_.

**Table 2.** ΔPmax, heart rate, and cardiac output responses to acute hypoxia: parameter values (mean ± SE) for the model of the cardiovascular response to hypoxia for the protocols with and without dexamethasone and with and without sustained hypoxia

	ΔPmax			Heart rate			Cardiac output		
Protocol	G mmHg/%	B mmHg	T min	G beats/min/%	B beats/min	T min	G l/min/%	B liters/min	T min
AM measurements									
*Protocol C-A*	0.66 ± 0.10	22.0 ± 1.2	1.8 ± 0.2	0.88 ± 0.07	60.0 ± 3.1	0.15 ± 0.12	0.09 ± 0.01	5.32 ± 0.31	0.30 ± 0.25
*Protocol C-H*	0.53 ± 0.06	22.6 ± 1.6	1.4 ± 0.5	0.88 ± 0.11	60.3 ± 1.6	0.78 ± 0.44	0.09 ± 0.01	5.50 ± 0.30	0.42 ± 0.29
*Protocol D-A*	0.52 ± 0.06	22.6 ± 1.9	1.8 ± 0.5	0.91 ± 0.12	62.1 ± 2.6	0.14 ± 0.08	0.08 ± 0.01	5.59 ± 0.20	0.15 ± 0.12
*Protocol D-H*	0.56 ± 0.07	22.0 ± 1.1	1.2 ± 0.4	0.89 ± 0.07	62.9 ± 3.8	1.19 ± 0.58	0.09 ± 0.00	5.80 ± 0.40	0.26 ± 0.14
PM measurements									
*Protocol C-A*	0.61 ± 0.09	22.1 ± 1.3	2.2 ± 0.5	0.85 ± 0.11	58.7 ± 3.0	0.38 ± 0.16	0.10 ± 0.01	5.07 ± 0.31	0.56 ± 0.33
*Protocol C-H*	0.72 ± 0.08	26.4 ± 1.4	0.8 ± 0.3	1.31 ± 0.16	68.3 ± 2.9	0.37 ± 0.14	0.13 ± 0.01	5.94 ± 0.35	0.32 ± 0.15
*Protocol D-A*	0.43 ± 0.05	25.7 ± 1.9	0.8 ± 0.4	1.22 ± 0.08	69.6 ± 2.0	0.76 ± 0.42	0.12 ± 0.02	6.72 ± 0.33	0.51 ± 0.33
*Protocol D-H*	0.69 ± 0.06	26.4 ± 1.2	1.3 ± 0.3	1.28 ± 0.15	85.5 ± 3.9	1.05 ± 0.34	0.11 ± 0.02	7.71 ± 0.48	0.75 ± 0.30
PM-AM measurements									
*Protocol C-A*	−0.05 ± 0.03	0.1 ± 0.5	0.4 ± 0.6	−0.03 ± 0.11	−1.2 ± 1.0	0.23 ± 0.19	0.01 ± 0.01	−0.25 ± 0.15	0.25 ± 0.47
*Protocol C-H*	0.19 ± 0.06*	3.7 ± 1.0**	−0.6 ± 0.5	0.43 ± 0.22	7.9 ± 3.2*	−0.41 ± 0.50	0.04 ± 0.02	0.44 ± 0.29	−0.09 ± 0.29
*Protocol D-A*	−0.10 ± 0.07	3.1 ± 0.6†	−1.0 ± 0.6	0.31 ± 0.12*	7.5 ± 3.1*	0.62 ± 0.44	0.04 ± 0.03	1.13 ± 0.25†	0.37 ± 0.36
*Protocol D-H*	0.13 ± 0.03**	4.3 ± 0.9†	0.1 ± 0.6	0.38 ± 0.16	22.6 ± 5.8†	−0.14 ± 0.44	0.02 ± 0.02	1.91 ± 0.33†	0.48 ± 0.39

ΔPmax, maximum pressure difference across tricuspid valve during systole; G, sensitivity of ΔPmax, heart rate and cardiac output to a reduction in saturation; B, baseline value for ΔPmax, heart rate and cardiac output before the onset of hypoxia and following relief of hypoxia; T, time constant from speed of response for the change in ΔPmax, heart rate, and cardiac output at the onset and relief of hypoxia. One-sample *t*-test conducted on the differences between the morning and afternoon measurements,

* *P* < 0.05;

† *P* < 0.01.

### Cardiovascular Responses to Hypoxia

**Fig. 2.**
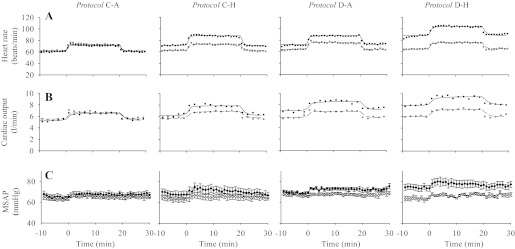
Mean values for heart rate (*A*), cardiac output (*B*), and mean systemic arterial blood pressure (*C*, mean ± SE) in the morning (AM measurements, open symbols or broken lines) and afternoon (PM measurements, closed symbols or continuous lines) in all four protocols. Lines through data for HR or CO indicate the fit of the cardiovascular model to the data.

Figure 2, *A* and *B* shows mean values for HR and CO, respectively, during the AM and PM acute hypoxic exposures for all four protocols. The parameters obtained from fitting the cardiovascular model to HR and CO together with results from the statistical comparisons are shown in Table 2. For the parameters for HR and CO from the model, no significant differences were found between the PM and AM values for *Protocol C-A*. In contrast, there were significant increases in baseline values of HR (B_HR_) for *Protocol C-H*, *Protocol D-A*, and *Protocol D-H*, while significant increases in baseline values for CO (B_CO_) were observed in *Protocol D-A* and *Protocol D-H*. There was no interaction between 8 h of isocapnic hypoxia and the administration of dexamethasone on B_HR_ and B_CO_. The sensitivity of HR responses to hypoxia was significantly increased only in *Protocol D-A*.

Values for blood pressure in each protocol are shown in Fig. 2*C*. Table 3 shows the average SAP in euoxia and in hypoxia, and the increase from euoxia to hypoxia. There were no significant differences between the PM and AM blood pressures in euoxia and hypoxia in *Protocol C-A* and *Protocol C-H*. In contrast, there were significant increases in blood pressure in euoxia and hypoxia in *Protocol D-A* and *Protocol D-H*. Only the increases from euoxia to hypoxia in AM and PM in *Protocol D-A* differed significantly from each other. There was no interaction between 8 h of isocapnic hypoxia and the administration of dexamethasone on the blood pressures in euoxia and hypoxia.

**Table 3.** Response of mean systemic arterial pressure to hypoxia for protocols with and without dexamethasone and with and without sustained hypoxia

Protocol	Euoxia (mmHg)	Hypoxia (mmHg)	Increase (mmHg)
AM measurements			
*Protocol C-A*	63.8 ± 1.9	65.5 ± 1.6	1.7 ± 0.8
*Protocol C-H*	63.3 ± 2.3	65.3 ± 2.5	2.0 ± 1.0
*Protocol D-A*	67.2 ± 1.3	67.8 ± 1.2	0.6 ± 0.9
*Protocol D-H*	63.8 ± 1.6	67.1 ± 0.9	3.3 ± 1.2
PM measurements			
*Protocol C-A*	65.2 ± 2.2	67.7 ± 2.0	2.4 ± 0.4
*Protocol C-H*	68.0 ± 3.0	71.8 ± 3.5	3.7 ± 1.0
*Protocol D-A*	69.4 ± 1.2	73.5 ± 1.4	4.1 ± 0.9
*Protocol D-H*	74.3 ± 2.7	78.2 ± 3.4	3.9 ± 1.4
PM-AM measurements			
*Protocol C-A*	1.4 ± 1.4	2.1 ± 0.9	0.7 ± 0.8
*Protocol C-H*	4.7 ± 2.0	6.5 ± 3.1	1.8 ± 1.7
*Protocol D-A*	2.2 ± 0.7*	5.7 ± 1.1†	3.5 ± 1.1*
*Protocol D-H*	10.5 ± 2.0†	11.1 ± 2.9†	0.6 ± 1.5

Values are means ± SE. One-sample *t*-test conducted on the differences between the morning and afternoon measurements.

* *P* < 0.05;

† *P* < 0.01.

### Plasma EPO Concentrations

**Fig. 3.**
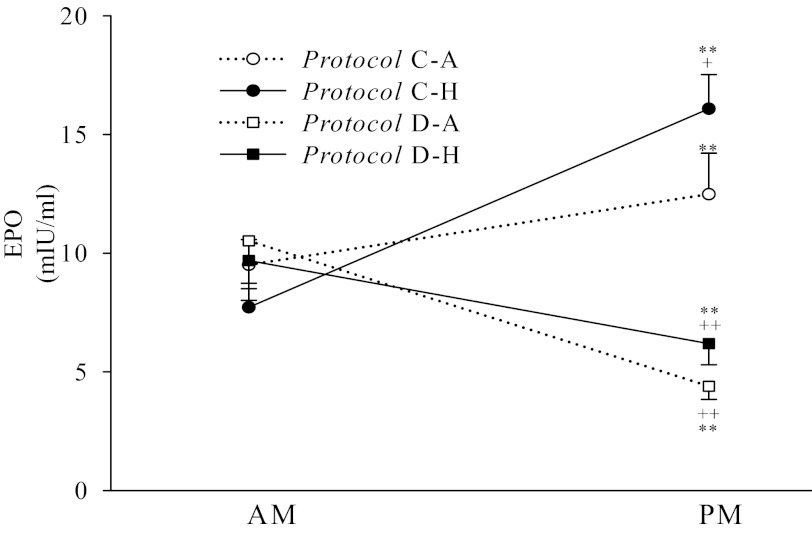
Plasma EPO concentration (mean ± SE) in each protocol. *Protocol C-A*, placebo and 8 h of air breathing; *Protocol C-H*, placebo and 8 h of isocapnic hypoxia; *Protocol D-A*, dexamethasone and 8 h of air breathing; and *Protocol D-H*, dexamethasone and 8 h of isocapnic hypoxia. AM, morning; PM, afternoon. Dexamethasone significantly decreased plasma EPO concentrations, whereas sustained hypoxia significantly increased plasma EPO concentrations. Dexamethasone also abolished the increase in plasma EPO concentration associated with sustained hypoxia. **P < 0.01 compared with the corresponding AM values; +*P* < 0.05, ++*P* < 0.01 compared with the PM values in *Protocol C-A*.

AM and PM plasma EPO concentrations are shown for the four protocols in Fig. 3. Dexamethasone administration significantly decreased the plasma EPO concentration over the course of the day, whereas hypoxia significantly increased the plasma EPO concentration.

ANOVA revealed no significant differences between the AM values for the protocols. On the whole dataset, there were significant effects of hypoxia by time, and drug by time, with no other terms being significant. This indicates that effects of dexamethasone and hypoxia are independent of each other.

## DISCUSSION

The main findings of this study were: *1)* dexamethasone mimicked sustained hypoxia with regard to increasing V̇e and ventilatory sensitivity to hypoxia; *2)* dexamethasone increased ΔPmax, HR, CO, and SAP in euoxia; *3)* dexamethasone and 8 h of hypoxia had additive effects upon V̇e, HR, and CO; *4)* dexamethasone decreased the expression of EPO and also prevented the increases in the expression of EPO resulting from 8 h of hypoxia.

We hypothesized that dexamethasone might enhance the beneficial ventilatory acclimatization to hypoxia while obtunding the disadvantageous pulmonary vascular response to hypoxia. The findings confirm the first part of this hypothesis while countering its second part. The pulmonary vascular response to 20 min of hypoxia following sustained hypoxia plus dexamethasone (*Protocol D-H*) was indistinguishable from that following sustained hypoxia alone (*Protocol C-H*).

How may we interpret these findings in relation to actual altitude exposure? There are three substantial relevant differences between true altitude exposure to hypoxia and the laboratory exposure to hypoxia used here, apart from the limited duration of these experiments. The first is that altitude exposure is hypobaric, rather than the normobaric exposure used in this study. Possible differences in physiological responses to these two kinds of hypoxia continue to raise debate (31, 32). The second is that altitude exposure is to a fixed, reduced inspired oxygen partial pressure, rather than the clamped Pet_O_2__ used here. The third is that altitude exposure involves hypocapnia; this has been avoided here by maintaining normal Pet_CO_2__. The increased V̇e and ventilatory sensitivity to hypoxia associated with dexamethasone in this study would be expected during altitude exposure to lower pulmonary artery pressure by alleviating the alveolar hypoxia and increasing the associated alveolar hypocapnia (1, 8). Thus, the study findings suggest that a stimulation of ventilation by dexamethasone at altitude may be the mechanism for a dual benefit in the prevention and treatment of high altitude disease: *1)* an improvement in systemic (and therefore cerebral) arterial oxygenation (45); and *2)* a reduction in pulmonary artery pressure arising from an elevation in Pet_O_2__ and a reduction in Pet_CO_2__.

### Effect of Dexamethasone on the Ventilatory Response to Hypoxia

Plasma dexamethasone concentrations reach a maximum around 2 h after oral administration (29), yet we did not observe any changes in V̇e in euoxia or the ventilatory response to hypoxia 2–3 h after administration of dexamethasone. However, by ∼10 h after the first of two doses of dexamethasone, significant increases in V̇e in euoxia and ventilatory sensitivity to acute hypoxia were observed. The literature contains few data for comparison. Garber et al. (15) found that ventilation increased in neonatal rats after 13 days of treatment with dexamethasone. Joseph et al., observing both acute (4-h) and chronic (10-day) effects of dexamethasone on adult rats, found that ventilation was unchanged by both regimes, yet the ventilatory response to hypoxia was substantially reduced (by 32%) 4 h after a single dose (19). Liu et al. have shown in adult rats an inflammatory response in the carotid body in response to hypoxia lasting 1 day and an anti-inflammatory response during dexamethasone administration that completely obtunds the enhancement of the acute carotid body efferent nerve activity response to hypoxia that normally follows 7 days of chronic hypoxia (28). The recent observation in rats that the nonsteroidal anti-inflammatory drug ibuprofen can obtund the enhancement of the acute ventilatory response to hypoxia that normally follows 7 days of chronic hypoxia (34) does tend to suggest that the proventilatory effects of dexamethasone observed in the present study may be independent of its anti-inflammatory effects (35).

Few ventilatory measurements appear to have been made in humans when dexamethasone has been assessed for its effects on altitude sickness. Ferrazzini et al. (12) found no change in ventilation after 12–16 h of dexamethasone treatment at 4,559 m (8 mg, then 4 mg, 6 hourly), yet arterial oxyhemoglobin saturation rose more than in controls, an observation on saturation repeated by Fischler et al. (13) (using 8 mg, 12 hourly). In contrast, Bernhard et al. (3) found no effect of dexamethasone (4 mg, 12 hourly) on arterial saturation at 6–8 h and 20 h at 5,334 m. Likewise, Levine et al. found no effect of dexamethasone (4 mg, 6 hourly) on saturation in volunteers exposed in a hypobaric chamber to a simulated altitude of 3,700 m for 48 h (24). These studies were poikilocapnic, and observed the effects of dexamethasone on hypoxic ventilatory response in the presence of changing levels of hypoxia and hypocapnia. The present study observed the effects of a constant level of hypoxia alone. It is possible that the latency of the delayed increase in V̇e in euoxia and the ventilatory sensitivity to hypoxia after administration of dexamethasone can be attributed to the need for gene expression to take place before the effects of the drug are observed.

Ventilation increased in euoxia and acute hypoxia after 8 h of isocapnic hypoxia in this study. This is consistent with previous reports (6). Both 8 h of hypoxia and administration of dexamethasone increased V̇e in euoxia and ventilatory sensitivity to acute hypoxia separately. The combination of 8 h of sustained hypoxia and dexamethasone in the present study led to particularly high levels of ventilation. It should be emphasized that these effects of 8 h of hypoxia and administration of dexamethasone on V̇e were in association with eucapnic hypoxia, and that exposures to poikilocapnic hypoxia in the true altitude setting are not immediately comparable.

### Effect of Dexamethasone on Pulmonary Vascular Responses to Hypoxia

Few studies have investigated the effect of dexamethasone on the pulmonary vasculature. Fischler et al. (13) observed a quite striking reduction in systolic pulmonary artery pressure in 10 volunteers with a history of high altitude pulmonary edema spending 2 days at 4,559 m on dexamethasone therapy compared with control therapy in 9 such volunteers.

In the present study, acutely, administration of dexamethasone had no effect on pulmonary arterial pressure and pulmonary vascular sensitivity to hypoxia. Unlike the effect on ventilation, administration of dexamethasone did not eventually affect the pulmonary vascular sensitivity to hypoxia; it only increased the baseline pulmonary arterial pressure. In contrast to this, acute hypoxia causes a rise in pulmonary artery pressure. Sustained hypoxia further increases the sensitivity of the pulmonary vasculature to acute hypoxia (7, 8). The previously documented effects of acute and sustained hypoxia on pulmonary vasculature were clearly observed in the present study. Interestingly, the rise in euoxic pulmonary artery pressure brought about by dexamethasone in PM measurements (∼3 mmHg) is about fourfold greater than the rise that can be anticipated from a previous study (2) from the increase in CO passing through the lungs alone (∼1 liter/min). This suggests that dexamethasone in euoxia may have some pulmonary vasoconstrictor activity.

There is interest in how these responses to hypoxia are generated at a cellular and molecular level. Work is beginning to associate some causes of a delayed rise in pulmonary artery pressure with the HIF system. HIF-1α has been shown to play a pivotal role in mediating both the vasoconstriction and vascular remodeling observed during the pathogenesis of hypoxic pulmonary hypertension in mice (40). Pulmonary artery pressure was increased in patients with Chuvash polycythemia who have impaired HIF degradation (4), and the pulmonary arterial pressure response to mild and moderate hypoxia was much greater in these patients (41). The pulmonary arterial pressure response to moderate hypoxia was also raised in patients with an HIF-2α gain of function mutation (14). These reports indicate that HIF may be associated with setting the intensity of the pulmonary arterial pressure response to hypoxia. However, in these studies, HIF-1α heterozygously deficient rats, patients with Chuvash polycythemia, and patients with a HIF-2α gain-of-function mutation had chronic pulmonary hypertension, and the structure of pulmonary arteries was likely to be remodeled. Therefore, it is unclear whether the increase in the pulmonary artery pressure and high cardiopulmonary response to acute hypoxia in these studies are a direct result of the upregulation of HIF target gene expression or the remodeling of pulmonary structure. In a study by Stelzner et al. (43), pulmonary arterial pressure in rats was significantly increased in euoxia after 48 h of hypobaric hypoxic exposure, and the pulmonary arterial pressure was further increased when reexposed to 12% O_2_; dexamethasone had no effect on these changes in pulmonary arterial pressure. The results of the present study are consistent with the study by Stelzner et al. It remains an open question therefore, whether the rise in euoxic pulmonary artery pressure brought about by dexamethasone in PM measurements is brought about by an upregulation of HIF target gene expression. If it is, it remains surprising that the acute pulmonary vascular response to hypoxia is not also enhanced in the PM measurements. Intermediaries that deserve future attention in addressing the mechanism of a raised pulmonary artery pressure in euoxia associated with dexamethasone include its effects on nitric oxide activity and water handling, locally in the lung, and in the body as a whole (30).

### Effect of Dexamethasone on Cardiovascular Responses to Hypoxia

The healthy humans in the study by Sambhi et al. (37) were given different glucocorticoids in different doses, and CO, HR, and SAP were measured within 3 h. The authors found that CO increased, although HR and SAP did not change, regardless of which kind of glucocorticoid was given and what dosage was given. In the study by Whitworth et al. (46), administration of dexamethasone 8 mg/d for 5 days significantly increased SAP and decreased HR in normal humans. In the present study, acutely, administration of dexamethasone had no effect on HR, CO, and SAP, but there were significant increases in HR, CO, and SAP about 10 h after the first dose of dexamethasone. It is known that patients with Cushing syndrome usually have high SAP (47). Some other studies have also observed that cortisol significantly increases SAP (20, 48). These results indicate that acute and chronic glucocorticoid administrations have different effects on HR, CO, and SAP.

In this study, 8 h of hypoxia significantly increased CO and HR, but did not significantly affect their hypoxic sensitivities. This is consistent with previous reports (5, 7). Mean SAP increased after 8 h of hypoxia, but the increase did not reach statistical significance. This is consistent with the study by Gilmartin et al. (16), in which no significant increase in mean arterial pressure was found following 8 h of poikilocapnic hypoxia.

### Effect of Dexamethasone on Expressions of HIF Target Genes

Few reports have investigated the effect of glucocorticoids on the expression of EPO. Zhang et al. (49) found that serum EPO decreased to undetectable levels in Sprague-Dawley rats and Wistar rats after administration of adrenocorticotropin hormone for 13 days. In fetal sheep, expression of EPO mRNA was significantly increased in kidney after bilateral adrenalectomy, and this change was reversed by cortisol infusion for 48 h (27). Dexamethasone treatment of pregnant ewes for 48 h could decrease expression of EPO mRNA in fetal kidney, but not the adult (27). Thus this evidence from nonhuman studies suggests that glucocorticoids may have an inhibitory effect on EPO gene expression. A human study by Kelly et al. (20) found the opposite effect, with high-dose cortisol (200 mg/day) increasing serum EPO concentrations significantly after 5 days, whereas low-dose cortisol (80 mg/day) failed to show an effect. The present study shows an inhibitory effect of dexamethasone on EPO concentrations.

Eight hours of isocapnic hypoxia increased plasma EPO concentration in this study. This is consistent with previous studies in which expression of EPO has been found to increase in humans exposed to simulated high altitude (10, 21). A striking finding in the present study is that administration of dexamethasone suppressed the increase in plasma EPO concentration caused by hypoxia. Others have found that, in HeLa cells, under hypoxic conditions, the expression of HIF-1 target genes was further upregulated by glucocorticoids via the glucocorticoid receptor (GR); this upregulation cannot be achieved by the other steroid receptors and is suggested to result from the interaction between the GR and the transactivation domain of HIF-1α (22). There is evidence supporting both an upregulation of HIF-1-dependent gene expression by the GR (22) and a downregulation (44), as well as the added complexity of the GR itself being upregulated by hypoxia acting via the HIF system (23).

A further level of interaction between dexamethasone, EPO expression, and responses to hypoxia is known from the rat studies by Soliz et al., which suggest that EPO stimulates breathing at both the level of the brainstem and the carotid body via actions at a specific EPO receptor (42). If such modulation is present in humans in the time scale of the present study, we would expect it to be obtunding the rise in ventilation associated with dexamethasone because of the drug's action in reducing the EPO concentration in the PM measurements.

### Conclusion

Dexamethasone enhanced the ventilatory acclimatization to 8 h of hypoxia while inhibiting the increase in serum EPO concentration caused by 8 h of hypoxia. It seems likely that the stimulation of ventilation in hypoxia is one mechanism whereby dexamethasone can contribute to a reduction in symptoms of acute mountain sickness.

## GRANTS

This study was supported by the Wellcome Trust.

## DISCLOSURES

No conflicts of interest, financial or otherwise, are declared by the author(s).

## AUTHOR CONTRIBUTIONS

Author contributions: C.L., K.L.D., and P.A.R. conceived and designed research; C.L., Q.P.C., S.K., J.T.B., M.H., T.G.S., K.L.D., and P.A.R. performed experiments; C.L. and P.A.R. analyzed data; C.L., K.L.D., and P.A.R. interpreted results of experiments; C.L. and P.A.R. prepared figures; C.L., K.L.D., and P.A.R. drafted manuscript; C.L., Q.P.C., S.K., J.T.B., M.H., T.G.S., K.L.D., and P.A.R. edited and revised manuscript; C.L., Q.P.C., S.K., J.T.B., M.H., T.G.S., K.L.D., and P.A.R. approved final version of manuscript.
